# Variant Library Annotation Tool (VaLiAnT): an oligonucleotide library design and annotation tool for saturation genome editing and other deep mutational scanning experiments

**DOI:** 10.1093/bioinformatics/btab776

**Published:** 2021-11-16

**Authors:** Luca Barbon, Victoria Offord, Elizabeth J Radford, Adam P Butler, Sebastian S Gerety, David J Adams, Hong Kee Tan, Andrew J Waters

**Affiliations:** Cancer, Ageing and Somatic Mutation Programme, Wellcome Sanger Institute, Hinxton, Cambridge, CB10 1SA, UK; Cancer, Ageing and Somatic Mutation Programme, Wellcome Sanger Institute, Hinxton, Cambridge, CB10 1SA, UK; Human Genetics Programme, Wellcome Sanger Institute, Hinxton, Cambridge CB10 1SA, UK; Department of Paediatrics, University of Cambridge, Cambridge CB2 0QQ, UK; Cancer, Ageing and Somatic Mutation Programme, Wellcome Sanger Institute, Hinxton, Cambridge, CB10 1SA, UK; Human Genetics Programme, Wellcome Sanger Institute, Hinxton, Cambridge CB10 1SA, UK; Cancer, Ageing and Somatic Mutation Programme, Wellcome Sanger Institute, Hinxton, Cambridge, CB10 1SA, UK; Human Genetics Programme, Wellcome Sanger Institute, Hinxton, Cambridge CB10 1SA, UK; Cancer, Ageing and Somatic Mutation Programme, Wellcome Sanger Institute, Hinxton, Cambridge, CB10 1SA, UK

## Abstract

**Motivation:**

CRISPR/Cas9-based technology allows for the functional analysis of genetic variants at single nucleotide resolution whilst maintaining genomic context. This approach, known as saturation genome editing (SGE), a form of deep mutational scanning, systematically alters each position in a target region to explore its function. SGE experiments require the design and synthesis of oligonucleotide variant libraries which are introduced into the genome. This technology is applicable to diverse fields such as disease variant identification, drug development, structure–function studies, synthetic biology, evolutionary genetics and host–pathogen interactions. Here, we present the Variant Library Annotation Tool (VaLiAnT) which can be used to generate variant libraries from user-defined genomic coordinates and standard input files. The software can accommodate user-specified species, reference sequences and transcript annotations.

**Results:**

Coordinates for a genomic range are provided by the user to retrieve a corresponding oligonucleotide reference sequence. A user-specified range within this sequence is then subject to systematic, nucleotide and/or amino acid saturating mutator functions. VaLiAnT provides a novel way to retrieve, mutate and annotate genomic sequences for oligonucleotide library generation. Specific features for SGE library generation can be employed. In addition, VaLiAnT is configurable, allowing for cDNA and prime editing saturation library generation, with other diverse applications possible.

**Availability and implementation:**

VaLiAnT is a command line tool written in Python. Source code, testing data, example input and output files and executables are available (https://github.com/cancerit/VaLiAnT) in addition to a detailed user manual (https://github.com/cancerit/VaLiAnT/wiki). VaLiAnT is licensed under AGPLv3.

**Supplementary information:**

Supplementary data are available at *Bioinformatics* online.

## 1 Introduction

The increase in human whole genome and whole exome datasets has led to the identification of millions of genetic variants. For example, the gnomAD consortium dataset contains sequence data for ∼200 000 individuals with close to 250 million high-quality variants (Karczewski *et al.*, 2020; Lek *et al.*, 2016). Most genetic variants are infrequent, with phenotypes in carriers influenced by a range of factors including the haplotype and genomic context in which the variant resides and the influence that an individual variant has on gene and/or protein function. Additional confounders include the variable expressivity of disease phenotypes and their penetrance, with these factors collectively reducing the precision with which variants can be conclusively interpreted (Starita *et al.*, 2017; Weile and Roth, 2018). Furthermore, individuals with previously unobserved variants at known disease-risk loci face an uncertain genetic diagnosis if they carry alleles not obviously deleterious, with novel missense variants routinely categorized as ‘Variant of Uncertain Significance’(Cooper, 2015). Therefore, understanding the functional impact of variants is a key challenge of modern genomics research with implications for clinical management and our fundamental biological understanding of disease genes. This challenge has been met in part through the *in vitro* functional assessment of discrete, often clinically observed, variants through assays and cell culture experiments. However, the rate of discovery of new variants in disease loci calls for a proactive approach to functional assessment using high-throughput, multiplexed assays of variant effects (MAVEs). MAVEs can assess coding and non-coding loci through approaches such as deep mutational scanning (DMS) and massively parallel reporter assays (MPRAs), respectively (Starita *et al.*, 2017). A range of DMS and MPRA technologies have been developed to produce variant effect maps, including yeast complementation (Hietpas *et al.*, 2011; Starr *et al.*, 2020; Sun *et al.*, 2016), cell display assays (Forsyth *et al.*, 2013; Starr *et al.*, 2020), FACS-based screens (Matreyek *et al.*, 2018) including sort-Seq MPRA (Kinney *et al.*, 2010), RNA-Seq MPRA (Melnikov *et al.*, 2012) and CRISPR/Cas9-based saturation genome editing (SGE) in human cells (Findlay *et al.*, 2018; Meitlis *et al.*, 2020). A distinct benefit of SGE is that variants are assessed within their endogenous genomic context, allowing interrogation of complex mutational consequences, including splicing effects. This increases the relevance of these data for clinical interpretation.

In a typical cell culture SGE experiment, a single guide RNA (sgRNA) complexed with Cas9, targets a genomic region to be edited and induces a DNA double-strand break (DSB). The presence of co-transfected variant-harbouring repair templates leads to the incorporation of nucleotide changes at the locus through homology directed repair (HDR). Insertions and deletions (indels) will likely also occur as a result of the non-homologous end joining (NHEJ) pathway of repair. The cell population is then phenotyped, most commonly by assessing the fitness/growth of edited cells, and then analysed using deep amplicon sequencing.

Whilst the concept of SGE is straightforward, the design and production of variant libraries is not a trivial process. To facilitate the use of SGE for diverse applications, we present a computational Variant Library Annotation Tool (VaLiAnT) that allows straightforward variant library design and *in silico* generation.

## 2 Materials and methods

### 2.1 Overview—SGE functionality

**Fig. 1.**
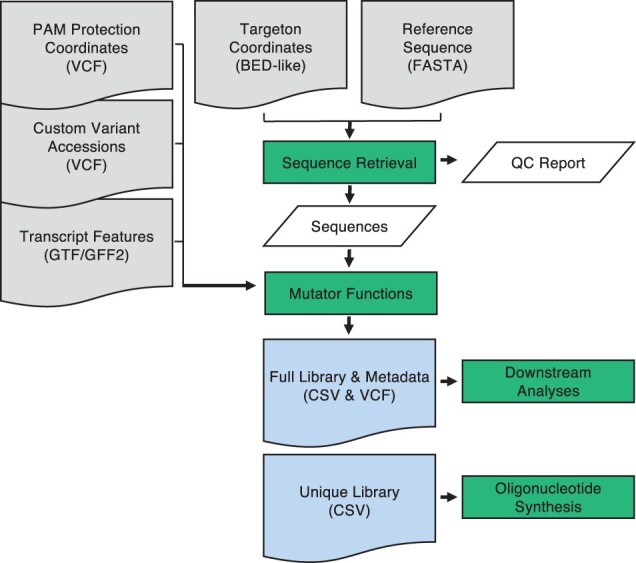
Information flow diagram for VaLiAnT: input files are shown as grey document boxes with file types shown in brackets. Processes are shown as green boxes and output files are shown as blue document boxes with file extensions in brackets. Process data is shown in slanted white boxes. Quality control (QC) report contains sequences in a comma-separated value (CSV) file and is execution-specific. All other output files are targeton-specific, see user-manual: https://github.com/cancerit/VaLiAnT/wiki. Abbreviations: Variant Call Format (VCF), Browser Extensible Data (BED), General Transfer Format (GTF), General Feature Format 2 (GFF2) and Fast-all (FASTA)

**Fig. 2.**
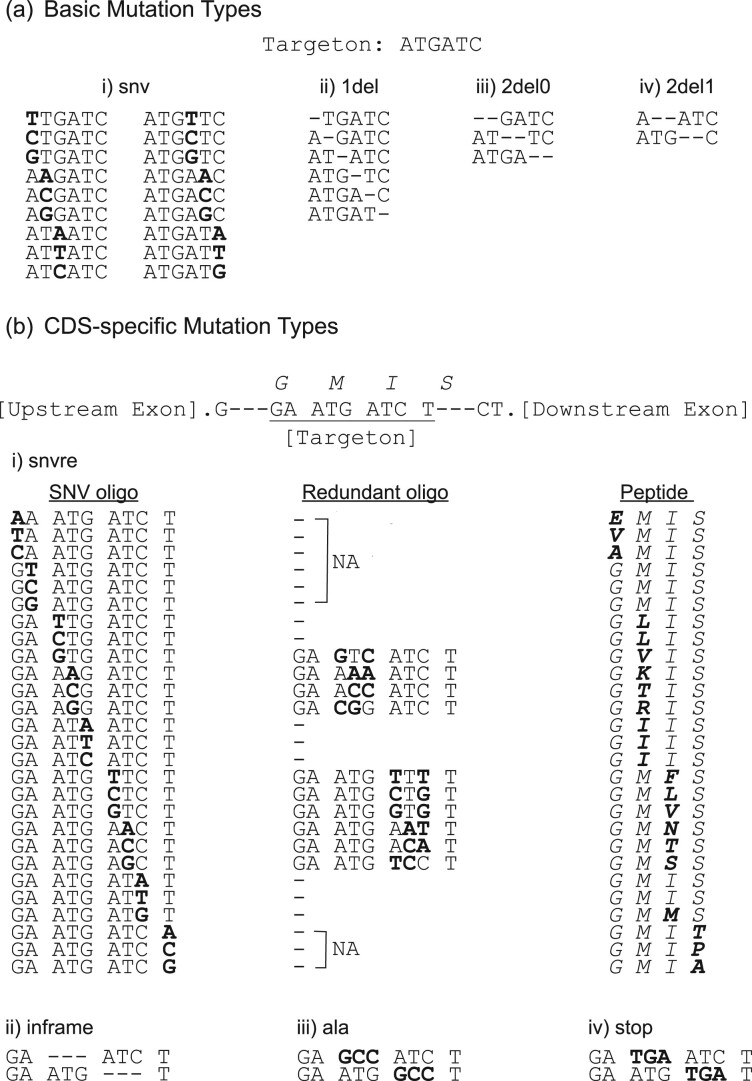
Mutator function descriptions: (**a**) Basic mutation actions that do not require CDS reading frame information. An example six base-pair targeton is shown for explanation purposes; (a-i) ‘snv’ results in all possible nucleotide substitutions at each position in the targeton, (a-ii) ‘1del’ deletes single nucleotides at each position, (a-iii) ‘2del0’ deletes nucleotides in tandem starting at position 0, (a-iv) ‘2del1’ deletes nucleotides in tandem starting at position 1. (**b**) Mutation actions that require CDS reading frame information. An example nine base-pair targeton is shown in which the first and last codon are split between upstream and downstream hypothetical exons. Amino acids encoded by codons are displayed in capital italics above the DNA sequence; (b-i) the ‘snvre’ mutation function runs the ‘snv’ function resulting in ‘SNV oligo’ output. CDS frame information is computed and where a missense change occurs as a result of ‘snv’—shown in ‘Peptide’ in bold—a ‘Redundant oligo’ is generated. This redundant oligo encodes the same missense change as that generated by ‘snv’ at the peptide level, but with an alternative triplet sequence. The redundant triplet sequence chosen is the most frequent according to the codon frequency table (or next most frequent if ‘snv’ generates the most frequent). Instances in which redundant oligos are not generated are represented by ‘–’ in the redundant oligo column, this includes; when synonymous changes are created by ‘snv’ (these are included in ‘snvre’ outputs), when ‘snv’ alone creates an additional missense change that results in an appropriate redundant oligo (as is the case for peptides GLIS and GIIS in the example), when non-degenerate missense codons are produced (ATG in the example) and when partial codons are targeted (denoted by ‘NA’; codons cannot be replaced, but SNVs are still introduced); (b-ii) ‘inframe’ results in codon, triplet deletions for the longest inframe coding sequence within the targeton; (b-iii) ‘ala’ results in inframe substitutions to alanine based on the top-ranking alanine from the codon usage table, resulting in an alanine scan through coding sequence; (b-iv) ‘stop’ results in inframe substitutions to a stop codon based on the top-ranking stop from the codon frequency table, giving systematic truncating mutations throughout the coding sequence

VaLiAnT is run from the command line. Inputs, outputs and broad processes are summarized in Figure 1 and Supplementary Figure S1. Reference files for genomic sequence (FASTA format), transcript features (GTF/GFF2 format) and custom variant accessions (VCF format) can be downloaded from repositories and stored locally. Multiple custom variant files can be used, with directories for VCF files listed in a custom variant manifest file (CSV format). Unique identifiers for the variant accessions will be extracted from the ‘ID’ column within the VCF files by default. Alternatively, unique identifiers can be parsed from user-specified INFO tags within the VCF files and included in the metadata outputs. Depending on the nature of the experiment, the user may wish to target exonic, intronic or a mixture of both exonic and intronic sequences for repair template generation. To communicate this, we use the term ‘targeton’ to describe the region of the genome that will be targeted. For each targeton reference sequence, different mutator functions may be applied (Fig. 2). Basic mutation types do not consider the coding sequence frame and are used for nucleotide-level saturation, actioned through ‘1del’, ‘2del0’, ‘2del1’ and ‘snv’ functions (Fig. 2a). CDS-specific mutation types consider the frame of the GTF/GFF2-specified transcript (Fig. 2b). Sitting between the nucleotide-level and protein-level saturation space, ‘snvre’ functions based on the amino acid-level mutational consequence (synonymous/missense/nonsense) of changes induced by ‘snv’ at the nucleotide-level (‘snvre’ includes the ‘snv’ function) (Fig. 2b–i). The purpose of ‘snvre’ is twofold. Firstly, to increase library representation of synonymous variants to facilitate downstream SGE analyses; as synonymous changes are unlikely to have a functional impact on protein function they can act as base-line negative controls for read-count normalization. When ‘snv’ creates a synonymous change at a codon due to triplet degeneracy, ‘snvre’ creates all possible synonymous codons at the same triplet position (‘snv’ alone creates all 2- and 4-fold degenerate codons, i.e. when a 1 base-pair change leads to all synonymous codons), expanding synonymous inclusion for 6-fold degenerate codons (i.e. where six different codons encode for each of arginine, leucine and serine) and stop codons (TGA>TAG and TAG>TGA). Secondly, when ‘snv’ creates a mutation that leads to a missense change, the wild-type codon is exchanged for the next most frequent triplet code for the same missense change (based on a default or user-defined codon frequency table), this allows for insights into the effect of codon sequence on missense changes. 

Protein-level saturation is actioned through the ‘aa’ function. This function exchanges each wild-type codon for the most frequent triplet code of each other amino acid (based on a default or user-defined codon frequency table). While the default codon frequency table is human, VaLiAnT allows the user to provide an alternative codon frequency table.

Oligonucleotide libraries that include both nucleotide-level and protein-level full saturation of average exon sizes are highly complex. This may limit coverage by amplicon sequencing to a desired level (with current SGE protocols) (Sakharkar *et al.*, 2000). Thus, VaLiAnT includes two functions as an alternative to the inclusion of full protein-level saturation sequences together with nucleotide-level libraries, namely ‘ala’ and ‘stop’ (Fig. 2b-iii and iv, respectively). These functions permit an inframe alanine scan through coding sequences, and the replacement of codons with stop codons, respectively. In addition, we have included a function, ‘inframe’ that removes codons to create inframe deletions (Fig. 2b-ii). Frame information for ‘aa’, ‘snvre’, ‘inframe’, ‘ala’ and ‘stop’ is derived from specific transcript GTF/GFF2 files provided by the user.

**Fig. 3.**
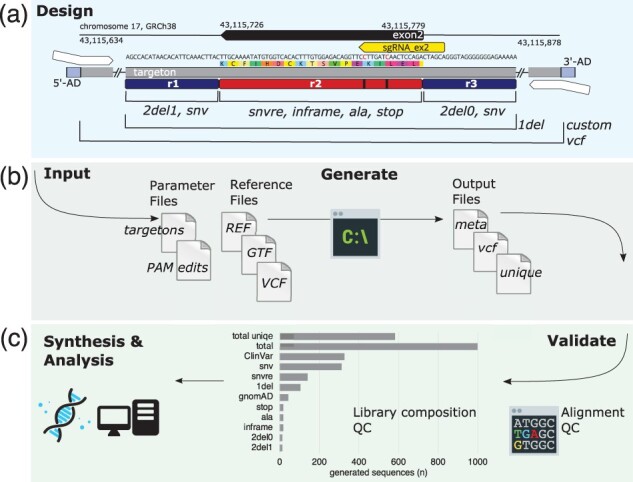
SGE library generation workflow for *BRCA1*: an example workflow for *BRCA1* exon 2 (*Homo sapiens*, GRCh38, ENSE00003510592) is shown with the corresponding input and output files used available from https://github.com/cancerit/VaLiAnT. (**a**) Overview of the library design, with sequence information modified from Geneious Prime^®^ (version 2019.04) visualization. Targeton, genomic region, chromosome and genome build are displayed, together with GRCh38 coordinates both for the complete targeton and for *BRCA1* exon 2. The selected sgRNA binding site is shown in yellow, where the directionality of the exon and sgRNA are negative strand and antisense, respectively. Within the nucleotide sequence, red nucleotides are positions selected for PAM/protospacer protection edits, beneath which the translated peptide sequence is shown by coloured rectangles. Below this, the targeton range is represented by a grey rectangle with condensed sequences represented by double slash. At either end of the targeton are the 5′-AD and 3′-AD (light blue rectangles) which represent appended P5 and P7 adapter sequences, enabling generic amplification of the generated library pool. Annealing sites for targeton-specific amplification and cloning adapters are shown as white arrows. Dark blue rectangles represent region 1 (r1) and region 3 (r3) and are 25 bp extensions from region 2 (r2), a red rectangle with black lines to indicate the location of the PAM/protospacer protection edits. Regions in which mutator functions are actioned are described through annotated black demarcation lines. Variants ingested through custom VCF files are incorporated throughout the entire range of the targeton. As shown, r1 and r3 are modified with basic mutation type functions. To ensure deletion of dinucleotide splice-acceptor or splice-donor intronic sequences immediately flanking exon 2, sequential deletion through r1(25 bp) is off-set using 2del1 (as r1 length is odd), enabling deletion of exon-flanking tandem nucleotides at the distal (3′) end of r1. As dinucleotide deletion proceeds from the 5′ end, 2del0 is used for r3, ignoring the final distal nucleotide of r3 at the 3′ end. r2 is modified by CDS-specific mutator functions. (**b**) Schematic of the input and output files used for computation. The ‘targetons’ file contains targeton and r2 genomic ranges, r1 and r3 extension values and additional information including the required mutator functions for each region and sgRNA identifiers which correspond with the identifiers given in the ‘PAM edits’ VCF file. Reference files include ‘REF’ FASTA chromosome sequence, ‘GTF’ specific transcript annotation, and a custom variant file ‘VCF’. VaLiAnT is run from the command line to generate output files, including ‘meta’ full library metadata, ‘VCF’ of all variants generated and a ‘unique’ csv file for easy ordering of sequence synthesis. (**c**) Downstream processes. Interrogation of the output files using sequence alignment is used for library validation. A library composition graph is shown, delineating each mutator function output for the entire targeton range of *BRCA1* exon 2, including total sequences and total unique sequences. Downstream synthesis, experimentation and/or analysis processes are possible after validation

The user is likely to require different mutator functions for intron sequence and exon sequence. VaLiAnT has the option to split targetons into sub-regions to allow for different mutator functions to be enacted within different ranges of the targeton (Fig. 3a). This information is defined by the user in the form of a BED-like input ‘parameter’ file. Targeton ranges are defined by 1-based indexing of genomic coordinates using ‘ref_start’ and ‘ref_end’ fields, with reference chromosome and strand also defined, as ‘ref_chr’ and ‘ref_strand’, respectively. Coordinates in fields ‘r2_start’ and ‘r2_end’ define a sub-region within the complete targeton range; typically, a complete or partial exon. A user-defined extension vector can then be applied to this range to define flanking sequence, upstream or downstream (relative to the positive strand) of r2. Sequence defined by r2 and sequence within the extensible 5′ and extensible 3′ ranges can be mutated independently by mutator functions. An action vector split into three components, specified in the targeton input file, designates mutator functions to be enacted on the distinct sequence ranges in respective order [5′extension (r1), r2, 3′extension (r3)]. Any targeton sequence defined in ‘ref_start’ to ‘ref_end’, but not in ‘r2_start’ to ‘r2_end’ or ‘ext_vector’, is included in oligonucleotide sequences as constant, unmodified genomic sequence (custom variants are still incorporated into constant regions). This is useful for defining flanking sequence for the annealing of downstream cloning adapters. In addition, generic adapters for universal amplification of all oligonucleotides in a synthesized pool can be specified on the command line and are appended after mutator function actions. The generic adapter feature can also be used to append cloning adapters if custom variant incorporation in cloning adapter sequence is not desired, but targetons need to be processed individually as this function appends to all sequences in the library. Any generated sequences exceeding a user-defined length (default > 300 bp) are excluded from standard output files with a warning displayed to the user and a separate metadata file returned.

To prevent Cas9 cleavage of variant sequences successfully incorporated at genomic loci, protospacer adjacent motif (PAM) and/or protospacer protection edits (synonymous or non-coding variants, refractory to Cas9 cleavage) can be included in sequence designs. sgRNA targets within the targeton are chosen by the user (selection parameters are outlined in Supplementary Table S1). Desired protection edits are also defined by the user, and inputted in the form of a parameter VCF file, in which position, reference and alternative nucleotide required are defined, along with an sgRNA identifier. The PAM and/or protospacer protection edits are incorporated into each targeton tagged with the sgRNA identifier in the targeton input file, with all sequences generated for that targeton containing the protection edits. More than one protection edit per sgRNA and/or targeton is possible.

### 2.2 Overview—cDNA DMS and prime editing saturation functionality

To create DMS libraries for cDNA studies, the ‘cdna’ function of VaLiAnT can be deployed. CDS-specific functions can be run on sequence supplied by the user to generate variant libraries. The ‘cdna’ feature requires a sequence input ‘cDNA targeton file’. This is a multi-FASTA file containing a desired CDS reference sequence. For a typical CDS experiment, the ‘cDNA targeton file’ may contain a CDS sequence within an expression vector. A ‘cDNA annotation file’ details targeton and mutagenesis range coordinates. Reading frame is determined based on user-defined coordinates. All mutator functions except ‘custom vcf’ are currently supported.

VaLiAnT can also be used to generate libraries for saturation prime editing (Erwood *et al.*, 2020). Prime editing works through the coupling of a Cas9 nickase to a Reverse Transcriptase (RT) domain (Anzalone *et al.*, 2019). A pegRNA (prime editing guide RNA) complexes with Cas9 and is directed to a locus, the pegRNA contains a Reverse Transcriptase Template (RTT) which includes a desired edit to a target sequence. The RT domain reverse transcribes the RTT, providing DNA template for nicked DNA repair. For VaLiAnT pegRNA construction the RTT is considered to be a targeton. Sequence is retrieved based on genomic coordinates in a ‘targeton input file’ and user-defined regions within the targeton are subjected to mutator functions to generate variant RTT sequences. ‘adapter-5’ and ‘adapter-3’ are used to append remaining pegRNA sequence. RTT directionality is dependent upon gRNA binding site directionality. VaLiAnT allows for this aspect to be included in pegRNA design through the ‘revcomp-minus-strand’ feature. All mutator functions including ‘custom vcf’ and PAM/protospacer protection edits are supported.

### 2.3 Implementation

The tool is implemented as a standalone executable Python package exposing a command line interface. Tabular data is managed via the Pandas package and interoperation with bioinformatic file formats via the PySam (FASTA and VCF) and PyRanges (GTF/GFF2) packages. The tool is species and genome-build agnostic.

### 2.4 Candidate region selection and sequence retrieval

PAM/protospacer protection edits are assigned via an sgRNA identifier to retrieved sequence defined by targeton range and used to create a modified reference sequence. The PAM/protospacer protected reference sequence obtained is the basis for subsequent mutations (custom or generated). Discrete mutation events are applied per oligonucleotide so that each generated sequence contains specific PAM/protospacer protection edits and the desired variant composed of one or more base changes.

#### 2.4.1 Targeton configuration

Targeton genomic ranges and desired mutator functions are detailed in a BED-like, tab-delimited file. Genomic ranges are expressed with the start position preceding the end position, regardless of the strand of the transcript. Each targeton can be divided into up to five regions (c1-r1-r2-r3-c2). Constant regions 1 and 2 (c1 and c2) are purposely not amenable to mutation through systemic mutator functions, but are still edited to include custom variants defined by VCF input. Regions 1-3 (r1-3) can be changed by mutator functions—each independently of each other—by detailing corresponding mutator lists in the BED-like input file. Both r1 and r3 ranges are derived through extension from the defined r2 range, detailed by a numeric list in the input file. Optional PAM/protospacer protection is configured via an sgRNA identifier list with corresponding variants retrieved from a dedicated VCF file.

#### 2.4.2 Reference annotation

To collect gene and transcript information and to apply CDS-specific mutator functions, appropriate transcript annotation must be provided via a GTF/GFF2 file; only CDS, UTR and stop features are taken into consideration. One transcript per gene is allowed to avoid ambiguities in matching target regions. No discrete target region (r1, r2, r3) within a targeton can span both coding and non-coding sequences; although the complete targeton can be divided into coding and non-coding regions (e.g. r1 and r3 could be non-coding and r2 coding). Constant regions (c1 and c2) can span coding and non-coding regions if desired. UTR sequences are processed in the same way as intronic sequences. Targeton region reading frame is computed using user-specified transcript feature annotations. Retrieval of additional positions from the reference sequence is necessary to obtain the context of partial codons. The frame of a sequence is obtained as follows (1), with *f* representing the frame as the number of bases missing from the codon at the 5′ end of a sequence, and *s* the start position:

(1) $$f_{\mathrm{target}}=f_{\mathrm{exon}}+(s_{\mathrm{target}}-s_{\mathrm{exon}})\;\mathrm{mod}\;3,\;s_{\mathrm{target}}\geq s_{\mathrm{exon}}$$

The number of additional positions 5′ and 3′ ($0\leq l_{\mathrm{ext}}\leq 2$), respectively, are obtained as follows (2), with *l* being the sequence length:

(2) $$l_{\mathrm{ext},5'}=f_{\mathrm{target}}$$

 

$$l_{\mathrm{ext},3'}=(3-(l_{\mathrm{target}}+f_{\mathrm{target}})\;\mathrm{mod}\;3)\;\mathrm{mod}\;3$$

The extra bases required at either end of the target may come from the same or an adjacent exon (Supplementary Fig. S2).

#### 2.4.3 Reference sequence retrieval

Reference oligonucleotide sequences are retrieved from a local FASTA file. Unless a sequence starts at position one, the nucleotide preceding it is also retrieved; this is required to generate the REF and ALT fields of liminal variants in the output VCF file.

#### 2.4.4 PAM/protospacer protection

The PAM/protospacer protection function applies custom single nucleotide variants that will be shared among all oligonucleotides for a targeton. The variant parameters are provided via a VCF file, each with an allocated SGRNA INFO tag, allowing variant grouping by sgRNA. sgRNA identifiers are assigned to targetons for specific inclusion.

### 2.5 Variant generation and output files

SGE and cDNA DMS functions are mapped to separate subcommands: ‘sge’ and ‘cdna’. Library type is listed in the source field (‘src_type’) in metadata output files, this can aid coordinate interpretation, which is absolute (i.e. genomic location) for SGE/saturation prime editing and relative (i.e. reference sequence position) for cDNA.

#### 2.5.1 Coding and non-coding variant generation

Variant incorporation can require different steps depending on whether the target region is a protein-coding sequence (CDS) or not. Mutator functions that result in basic mutation types, namely SNVs, single and tandem nucleotide deletions, can be applied to either type of target. CDS-specific mutator functions that result in codon replacement or deletion are only applied to complete codons within CDS targets (partial, liminal codons are ignored). SNVs carry extra information when introduced into CDS targets (compared with non-CDS targets), namely whether the SNV results in a synonymous, missense or nonsense change. SNVs can be introduced into partial, liminal codons in addition to complete codons; the annotation of the mutations in partial codons is informed by the exonic bases adjacent to the target (i.e. the sequence of the upstream and downstream codons). Variants that require the annotation of amino acid changes and mutation type (synonymous, missense or nonsense) are generated from computed metadata tables. The number of possible codons with a single SNV is limited, namely three per base per codon per strand ($3\cdot 3\cdot 4^{3}\cdot 2=1152$). For the same codon on different strands, the nucleotide changes are the same but the amino acid changes, and therefore their classification (synonymous, missense or nonsense), differ. If a mutator that requires SNV annotation is selected (‘snv’ or ‘snvre’), a partial metadata table, indexed by codon, is computed at start-up based on the codon table for each strand represented in the inputs. Similar considerations apply to the metadata of ‘snvre’ and codon substitutions. A partial table of all synonymous codon substitutions can also be generated from which a partial table of the top-ranking synonymous codon substitutions is subsequently derived.

#### 2.5.2 Custom variants

Custom variant VCF files are defined in a manifest list file. Simple variants are currently supported, including substitutions, insertions, deletions and indels. To remain source agnostic, insertions and deletions are classified based on the POS, REF and ALT fields. Start positions are corrected as appropriate before mapping variants to targetons. To preserve variant provenance, an alias is assigned to each VCF file. Optionally, an INFO tag mapping to an identifier (e.g. ‘ALLELEID’ for ClinVar variants) can also be assigned. By default, the VCF ID field is used as the variant identifier. Variants that start and end within the targeton are applied, generating one oligonucleotide sequence each.

#### 2.5.3 Oligonucleotide sequence output

Either three or four targeton-specific output files and one sequence quality control (QC) file (reporting retrieved wild-type sequence for the specified ranges) are generated per execution. The file names report chromosome, coordinates, strand and the sgRNA IDs associated with the targeton (Supplementary Tables S2 and S3).

Variant oligonucleotide sequences are generated by replacing target regions in PAM/protospacer protected reference sequence with a mutated sequence. The final oligonucleotide sequences are then assembled from: invariant region sequences (optional), adaptor sequences (optional) and target region mutated sequences. All variants are applied to the positive strand sequence. For negative strand transcripts, reverse-complemented final targeton sequence (excluding any adapter sequence) can be generated (optional) for ease of interpretation or for experimental requirement. The directionality of sequence for single-stranded synthesis will not affect downstream SGE experiments if PCR amplification (resulting in double-stranded DNA HDR template) forms part of library processing. In the case of oligonucleotides exceeding a specified, configurable length (e.g. due to a long insertion variant), metadata is segregated and sequences are not included in the unique file (a fourth output file containing excluded sequence information is generated).

#### 2.5.4 Output files and formats

Supplementary Table S2 lists output files and their purpose. It should be noted that the output metadata format does not follow VCF convention in reporting positions, reference and alternative sequences to favour streamlining of downstream processing. These departures only apply to deletions and insertions:

- deletions start at the first deleted position, the reference sequence starts at the same position, no alternative sequence is reported;
- insertions start at the position following the last unaffected nucleotide, the alternative sequence starts at the same position, and has no reference sequence.

Reference and alternative sequences for variants starting at position one similarly do not include the nucleotide that immediately follows this position.

## 3 Results

### 3.1 *BRCA1* saturation genome editing libraries

As an exemplar of the workflow and output of VaLiAnT we have generated oligonucleotide libraries suitable for performing SGE experiments on the exons encoding the RING domain of the tumour-suppressor gene *BRCA1* (*Breast cancer type 1 susceptibility protein*). From the transcript NM_007294.4 (ENST00000357654.9, Gencode release v34), exons 2, 3, 4 and 5 are targeted. A summary of the process for exon 2 of *BRCA1* is shown in Figure 3. All input and output files are available from https://github.com/cancerit/VaLiAnT. Input coordinates consist of GRCh38 genomic ranges within *BRCA1* (ENSG00000012048.23, Ensembl release GRCh38.p12) for four targetons spanning each of exons 2, 3, 4 and 5 and PAM/protospacer protection edits at one sgRNA binding site per targeton. Input coordinates for exon 2 are shown in Figure 3a and input criteria for exon 2 shown in Table 1. sgRNAs and PAM/protospacer protection edits were selected based on design parameters outlined in Supplementary Table S1, with sgRNA sequence and off-target scores obtained from the Wellcome Sanger Institute Genome Editing database (WGE) CRISPR search tool: https://wge.stemcell.sanger.ac.uk/ (Hodgkins *et al.*, 2015). Targetons 2, 3 and 4 are 245 bp in length, covering the complete CDS of the exon with 25 bp extensible regions (r1 and r3) to target flanking intronic sequence. Targeton 5 is 251 bp in length with extensible regions of 20 bp (r1) and 41 bp (r3) at 5′ and 3′ of the exon, respectively. Nucleotide and amino acid saturation libraries have been created independently (brca1_nuc and brca1_pep, respectively), with all mutator functions except ‘aa’ represented in the nucleotide library and ‘aa’ and ‘inframe’ in the amino acid library. Custom variants using ClinVar [release 2020-11-07, (Landrum *et al.*, 2018)] and gnomAD [v3.0, (Karczewski *et al.*, 2020)] VCF files for *BRCA1* have been incorporated in brca1_nuc (VCF files were filtered for genomic coordinates of interest to reduce file sizes).

**Table 1.** Summary of input parameters for *BRCA1* exon 2 SGE library generation

Input parameter	Value
chromosome	17
strand	-
targeton range	43115634 : 43115878
r2 range	43115726 : 43115779
r1 length	25
r3 length	25
sgRNA identifier	sgRNA_ex2
mutators for r1	2del1, snv, 1del
mutators for r2	snvre, inframe, ala, stop, 1del
mutators for r3	2del0, snv, 1del

*Note*: Summary of values included in targeton parameter input file for exon 2. Values correspond to Figure  3a schematic.

**Table 2.** Summary of output sequence categories for *BRCA1* exon 2 SGE library generation

		r1	r2	r3	Constant	Complete
	Length (bp)	25	54	25	141	245
Mutator functions	2del1	12	–	–	–	12
	2del0	–	–	12	–	12
	1del	25	54	25	–	104
	inframe	–	17	–	–	17
	snv	75	162	75	–	312
	snvre	–	140	–	–	140
	ala	–	17	–	–	17
	stop	–	17	–	–	17
	gnomAD	2	8	10	22	42
	ClinVar	74	176	76	1	327

	Total	188	591	198	23	1000
	Excluded	0	1	0	0	1
	**Unique**	109	351	101	22	**583**

*Note*: Values for each attribute are shown, specific to regions 1–3 (r1–3) and constant regions (unedited, except for custom variants) and the summed values comprising the entire targeton (complete). One custom variant in r2 results in an oligonucleotide longer than 300 bp and is excluded from the final library, total unique oligonucleotides—the number representing library complexity for SGE experiments—is shown in bold. Values relate to targeton ‘chr17_43115634_43115878_minus_sgRNA_ex2’ (https://github.com/cancerit/VaLiAnT/tree/develop/examples/sge/brca1_nuc_output_exp).

Exon 2 library complexity is summarized in Table 2 with a summary of the complexity of each library provided in Supplementary Table S3. P5 and P7 sequences were selected as adapter sequences to be added 5′ and 3′ of each oligonucleotide generated. Sequences that exceed 300 bp were omitted from unique sequence files using the ‘max-length’ option. As *BRCA1* is transcribed from the negative strand, the ‘revcomp-minus-strand’ option was used to generate reverse complement sequence. This does not affect correct sequence generation, it is a preference for intuitively validating the sequences that are generated.

### 3.2 *BRCA1* cDNA deep mutational scanning library

VaLiAnT can also be used in a cDNA DMS library generation workflow to produce libraries for cassette expression studies. To demonstrate the utility of the cDNA library generation feature of VaLiAnT, we have designed a DMS library suitable for the doxycycline inducible expression of *BRCA1* with saturation of coding sequence variants. All steps are *in silico*. We took the canonical *BRCA1* transcript (ENST00000357654.9) and extracted the 5592 bp CDS (coding sequence), that encodes for protein NP_009225.1. A total of 22 exons are present. This CDS was inserted into pCW57.1 (addgene #41393) downstream of a Tetracycline Response Element (TRE) promoter (by *in silico* digestion of pCW57.1 with SalI and NehI), to enable hypothetical inducible expression of variant harbouring cDNA by doxycycline treatment after transduction. 40 targetons between 132 and 237 bp in length were designed to cover the 22 concatenated exons that encode NP_009225.1. CDS ranges—defined by ‘r2_start’ and ‘r2_end’ in the cDNA targeton file—were between 81 and 195 bp and were selected to cover all CDS sequence. Targetons and r2 ranges were tiled over larger exons such that some sequence between the two ranges overlaps (https://github.com/cancerit/VaLiAnT/wiki/Advanced-usage). The following mutator functions were run for each targeton: ‘snv’, ‘1del’, ‘snvre’, ‘ala’, ‘stop’, ‘inframe’, ‘aa’. The number of unique oligonucleotide sequences generated in each library ranged from 858 to 2092, with an average of ∼1600 nucleotides. The total number of variants to cover all of the CDS and making up the final concatenated library is 62 754. A detailed description, and input and output files are available here: https://github.com/cancerit/VaLiAnT/wiki/cDNA-example.

### 3.3 *BRCA1* prime editing saturation library

We have designed saturation prime editing libraries to saturate the coding sequence and flanking intronic sequence of *BRCA1* exon 2. The sequence is covered using two pegRNAs with variant harbouring RTT regions. pegRNA_a and pegRNA_b have variant RTT regions with lengths of 47 bp and 49 bp, respectively. Together the RTT regions cover the 54 bp CDS of *BRCA1* exon 2 and 4 bp flanking intron 5′ and 3′ of the exon. GRCh38 coordinates were entered into input targeton and PAM/protospacer protection files. ref_start and ref_end in the targeton file are coordinates for RTT start and RTT end. r2_start and r2_end in the targeton file are coordinates for a CDS-specific (frame-considered) range within the targeton range. A PAM protection and protospacer synonymous mutation were defined for pegRNA_a and a PAM protection synonymous mutation for sgRNA_b; mutations are in the r2 range for both pegRNA_a and pegRNA_b. The transcript GTF file (for ENST00000357654.9), genomic sequence reference file (FASTA and index), custom VCF manifest (and defined VCF files), are not changed from a standard SGE library design approach (https://github.com/cancerit/VaLiAnT/tree/main/examples/sge/reference_input_files).

The pegRNA_a RTT library contains 454 unique sequences, and the pegRNA_b 483. Additional pegRNA sequences necessary for prime editing were appended to variant RTT sequences through ‘adapter-5’ and ‘adapter-3’ functions. P5 and P7 sequences for generic amplification of the library were also included. The general structure of the pegRNAs can be summarized as follows; P5, Golden Gate 5′ adapter (Type IIS cohesive end), sgRNA spacer and pegRNA scaffold sequence were appended at the 5′ of variant RTT and primer binding site, Golden Gate 3′ adapter (Type IIS cohesive end) and P7 sequence were appended 3′. Sequences longer than 250 bp were excluded using the ‘max-length’ feature. All input and output files and a detailed construction description are available at https://github.com/cancerit/VaLiAnT/wiki/Saturation-prime-editing-example.

## 4 Discussion

As the technology to write DNA catches up with our ability to read DNA, having the ability to computationally generate multiplexed variant libraries from reference sequence is a critical starting point for many studies. We present VaLiAnT, a software package that is immediately useful for those who wish to perform SGE experiments. Furthermore, VaLiAnT is agnostic with respect to species, genome build, transcript and custom variant source. The definition of targeton sequence range and the delineation of sub-regions within the targeton is versatile and can be specified to suit experimental needs. Optional, user-defined PAM/protospacer protection edits required to prevent Cas9 (or alternative Cas protein) cleavage of incorporated HDR template at target loci can be incorporated into all targeton output sequences.

We have created a range of functions to introduce changes within oligonucleotide sequences which represent templates for SGE. Mutator functions within the software systematically edit sequence to allow investigation in both nucleotide and protein space. Functional assays normally involve interpretation of nucleotide- and protein-level change. For example, clinical interpretation of a single nucleotide variant that leads to a missense change inherently involves drawing conclusions about the effect of a change in the protein sequence. In terms of questions that can be addressed through deep mutational scanning, the relative importance of nucleotide-level and protein-level saturation can be roughly attributed. Clinical interpretation and prediction of variant effect, including splice sites, will mostly be informed by saturating at the nucleotide-level; fundamental biological investigation of structure-function relationships and drug target development will likely benefit most from saturation at the protein-level; and studies in pathogen and evolutionary biology will benefit from interpretation of both nucleotide-level and protein-level saturation data.

There are several reported methods to create nucleotide variant libraries using either error-prone PCR or forms of chemical synthesis. While error-prone PCR is comparatively cheap, variants cannot be systematically generated to the same degree as with oligonucleotide-array, chemical synthesis platforms. *In vitro* enzymatic generation of oligonucleotides using the template-less DNA polymerase terminal deoxynucleotidyl transferase is being pursued by several biotechnology companies with some encouraging developments, and might prove to be an effective alternative to chemical synthesis in the future (Liberante and Ellis, 2021). At the time of writing, chemical synthesis at the scale of ≤300 bp is possible on a large scale. Undoubtedly, as synthesis platform technology advances, an increase in oligonucleotide synthesis length (and reduction in error rate) will become possible. We have provided an optional filter to remove sequences above a user-defined length, which is configurable to accommodate for future increases in synthesis capability.

Although VaLiAnT has been principally designed to aid in the design and generation of oligonucleotide libraries for SGE experiments, its uses also include but are not limited to: mutational consequence annotation of coding-sequence variation, conversion of VCF annotation files to oligonucleotide sequences and the generation of cDNA DMS libraries for exogenous or orthogonal assays (as opposed to genome editing), that use cDNA cassettes and/or transcriptional readouts (Jones *et al.*, 2020; Ursu *et al.*, 2020).

The reverse-complement option in VaLiAnT permits the generation of variant sequences for strand-specific applications. For example, SGE using single-stranded oligodeoxynucleotides (ssODN) donor template has been reported to streamline experimental procedures, however the HDR rate 5′ and 3′ of the Cas9 cut site differs depending on ssODN orientation (Kan *et al.*, 2017; Meitlis *et al.*, 2020). VaLiAnT can be used to generate orientation-specific libraries for ssODN-based SGE experiments to maximize HDR rate.

Prime editing**-**based saturation mutagenesis is another strand-specific application in which VaLiAnT can be used. Reverse-transcriptase templates (RTTs), which contain the desired variants and are strand-specific, form part of prime editing guide RNAs (pegRNAs) (Anzalone *et al.*, 2019). VaLiAnT can be used to produce saturating edits in extensible RTT regions of pegRNAs and the adapter option used to append scaffold and guide RNA spacer sequence, producing pegRNA oligonucleotide sequences for synthesis and subsequent saturation mutagenesis. Guide selection and RTT window selection may be informed through use of pegFinder (Chow *et al.*, 2021) and/or PrimeDesign (Hsu *et al.*, 2021) tools.

Saturation genome editing (SGE) provides rich information for functional analysis of genetic variation as variants are assayed in their endogenous genomic environment. As SGE and other DMS studies with next-generation sequencing readouts increase in number, it is anticipated that tissue/cell-specific studies, will provide further context and biological relevance in addition to genomic environment. Tissue/cell specific analysis of variants and their effects on protein structure and protein-partner complexes is also an area that DMS will greatly inform. Inferring protein structure from DMS data is possible as is imputation of probable variant effects from a mutational spectrum (Dunham and Beltrao, 2021; Schmiedel and Lehner, 2019). In addition, as predicative models and machine learning increase in utility for the characterization of the properties of mutational change and the prediction of the likely effect of specific mutational changes, particularly in relation to protein-protein interactions, training datasets rich in biological context will be important (Senior *et al.*, 2019). Therefore, being able to use specific genomic reference sequence and distinct transcript information in the design of variant libraries—which VaLiAnT allows—will become desirable.

We envision that, as opensource software, VaLiAnT will be further modified by the community. For example, by the addition of new mutator actions to expand the repertoire of available functions useful for different forms of experimentation, annotation or analysis. There is also scope for VaLiAnT to be combined with other software, such as upstream heuristic functions to select appropriate input information, such as; targeton ranges, sgRNAs and PAM/protospacer protection edits. In addition, VaLiAnT may be combined with downstream analysis software tools for bioinformatic analysis of deep mutational scanning data, for example; to map functional readouts to variants within libraries and calculate statistical measures with pipelines/software such as DiMSum (Faure *et al.*, 2020), Enrich2 (Rubin *et al.*, 2017) and DESeq2 (Love *et al.*, 2014), to calculate functional scores of variants with bespoke scripts, and to produce annotated visualizations of variant effect.

VaLiAnT provides a novel way to systematically produce *in silico* variant libraries and corresponding annotations and metadata. We believe this software package will become an essential component of the deep mutational scanning toolkit, significantly contributing to the study of genetic perturbations at scale.

## Supplementary Material

btab776_supplementary_data
