# Supplementary material for: Variant Library Annotation Tool (VaLiAnT): an oligonucleotide library design and annotation tool for saturation genome editing and other deep mutational scanning experiments
Source: Bioinformatics. 2021 Nov 16;38(4):892–9. doi: 10.1093/bioinformatics/btab776 (PMC8796380; doi:10.1093/bioinformatics/btab776)
Supplement: btab776_supplementary_data [file btab776_supplementary_data.docx]

**Supplementary Material**

**
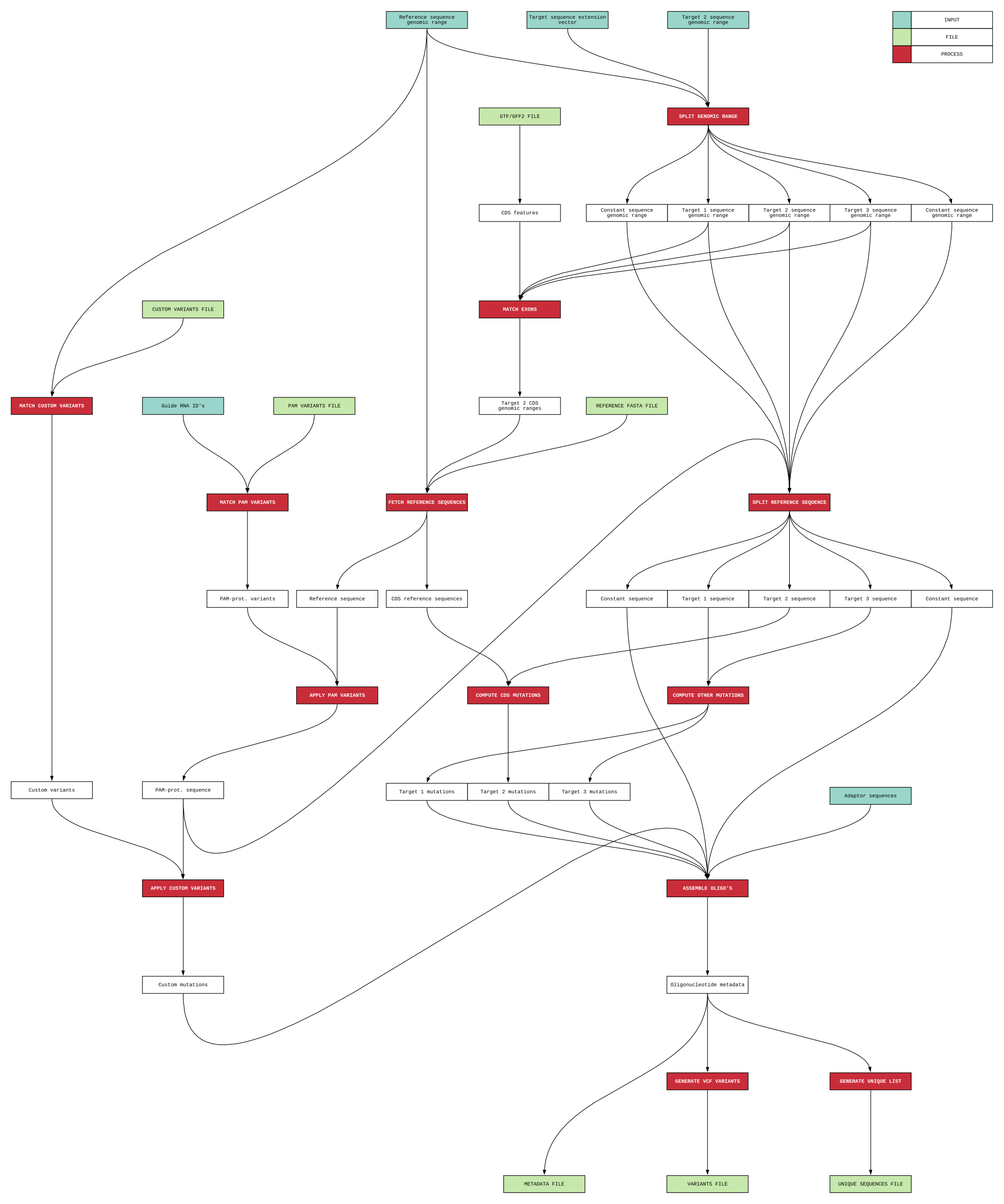
Variant Library Annotation Tool (VaLiAnT): an oligonucleotide library design and annotation tool for Saturation Genome Editing and other Deep Mutational Scanning experiments**

**Supplementary Fig. 1: Flow of information through VaLiAnt:** inputs (‘INPUT’) are represented by dark green rectangles, files (‘FILE’) by light green rectangles and processes (‘PROCESS’) by red rectangles.


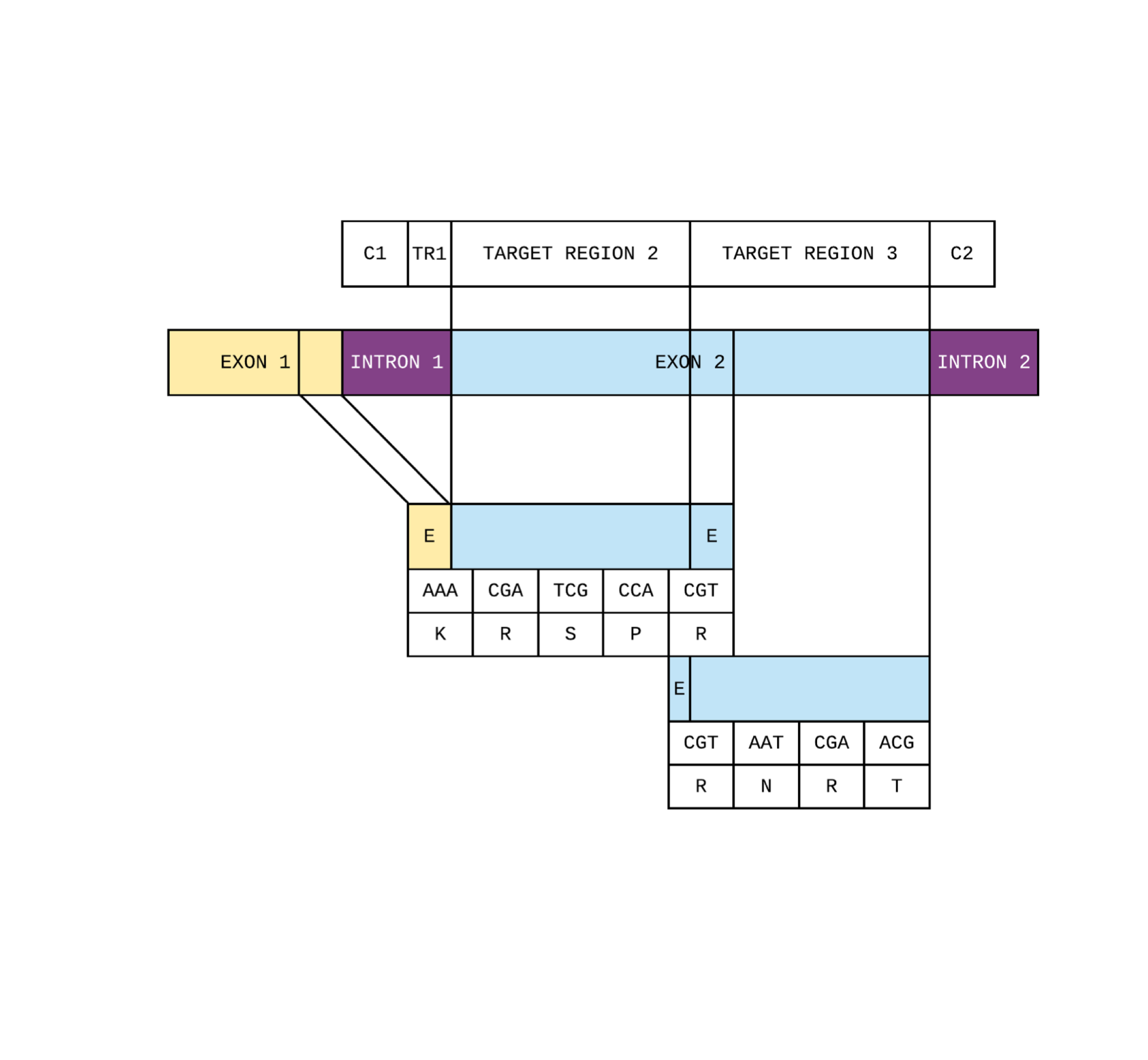


­

**Supplementary Fig. 2. Reading frame calculation logic:** an example targeton with the first constant (C1) and target (TR1) regions on the first intron of a transcript, the second and third target regions on the second exon, and the second constant region (C2) on the second intron. The 5’ CDS extension for TR2 is retrieved from the preceding exon (exon 1) and the 3’ CDS extension from the same exon targeted (exon 2). TR3 requires no CDS extension at 3’ because the second exon ends in-frame.

**Supplementary Table 1.** sgRNA site and PAM/protospacer protection nucleotide selection criteria

| sgRNA selection criteria | | PAM/protospacer protection criteria | |  |
| --- | --- | --- | --- | --- |
| *Attribute* | *Reason* | *Attribute* | *Reason* | |
| sgRNA binding site at appropriate locus. | Fits design parameters. | Only mutate 3^rd^ base of a codon at PAM site or in protospacer. | Codon redundancy allows for synonymous edits. | |
| sgRNA binding site within targeton CDS (if targeting exon). | Frameshifting indels created by NHEJ will deplete (if CDS mutation is deleterious) increasing relative representation of HDR edits in screen. | Always change pyrimidines to pyrimidines (C/T) and purines to purines (A/G). | Missense changes caused by SNV saturation at PAM/protospacer protected codons will replicate missense changes achieved as if codon were wild-type (with some exceptions*). | |
| Synonymous edit possible at PAM and/or protospacer. | Prevents Cas9 from targeting HDR library template, which depletes representation of edited loci. | Wherever possible select a pyrimidine base to change. | No exception to above missense representation. | |
| No 0 (exact), 1 or 2 mismatch to CDS off-target. | Avoids off-target effects and preserves on-target activity. | Wherever possible change terminal G of PAM over more protospacer proximal G. | Avoids NAG PAM sites which spCas9 still cuts with limited efficacy. | |
| No 4 (or more) consecutive thymine residues (TTTT). | Prevents premature transcriptional termination of sgRNAs expressed from RNA polymerase III promoters (such as U6::3). | Antisense sgRNAs are preferred (CCN PAM rather than NGG). | An increased number of pyrimidine changes are possible. | |
| Avoid sgRNAs that have G/GG adjacent to PAM NGG (leads to two consecutive GG at PAM after protection edits). | Despite PAM editing to protect from Cas9, cutting still occurs if NGG exists adjacent to wild-type PAM. | No protection edits in the unique codons, ATG[M] and TGG[W] or ATA[I]. | Purine protection edits at the 3^rd^ position of these codons do not lead to synonymous changes. | |

*in addition to a purine change at 3^rd^ base, if: 1^st^ base=T or 2^nd^ base=G, then W/STOP missing in SNV SGE library; 1^st^ base=A or 2^nd^ base=T, then M/I missing in SNV SGE library; 1^st^+2^nd^ base=TT or 1^st^+2^nd^ base=AG, then both W/STOP and M/I will be missing in SNV SGE library.

Summary of attribute considerations and reasons driving selection of sgRNAs for *BRCA1* exon 2-5 SGE library design and nucleotide selection for PAM/protospacer protection edits within the sgRNA binding site sequence.

**Supplementary Table 2.** Summary of output file nomenclature and use

| Filename | Description | Purpose | Targeton-specific | Execution-specific |
| --- | --- | --- | --- | --- |
| _meta.csv | Metadata and sequence annotation file | Bioinformatic analyses | **✓** | – |
| _meta_excluded.csv | Metadata file for sequences above specified length | QC of sequence exclusion | **✓** | – |
| _unique.csv | Filtered file containing only unique sequences and oligo name | Synthesis submission | **✓** | – |
| _.vcf | Variant Call Format of generated and custom variants | Bioinformatic analyses | **✓** | – |
| ref_sequences.csv | Retrieved sequences for targetons and sub-regions | QC of sequence retrieval | – | **✓** |

Details are shown for the five possible output files for a VaLiAnT processed targeton. For each output file, ‘filename’ shows the final descriptive extension appended to the targeton file names (see <https://github.com/cancerit/VaLiAnT> for the *BRCA1* exon 2 example output files). Broad and non-exclusive ‘purposes’ are noted. All files except the ‘ref_sequence.csv’ sequence retrieval QC file (which is produced per execution and contains information on all inputted targetons) are generated per targeton. Exclusion metadata files are only produced when a generated sequence exceeds 300 bp (default) or a user-specified maximum length.

**Supplementary Table 3.** Summary of library complexity for generated *BRCA1* libraries

**‘**library’ name corresponds to exon and saturation space, ‘generated name’ contains targeton-specific generated nomenclature to describe library parameters, ‘saturation space’ is either nucleotide or amino acid-level, nucleotide mutator functions are as described in Fig. 3a for each of exon 2, 3, 4 and 5. Amino acid-level contains only ‘aa’ and ‘inframe’ mutator functions directed at the same targeton ranges and regions as the nucleotide libraries. ‘targeton length’ is the length in base-pairs of the entire targeton, ‘saturation length’ is the length in base-pairs of the sequentially mutated regions within the targeton (that is r1, r2, r3 combined), ‘total sequences’ is the number of oligonucleotide sequences produced (including multiples of identical sequence), ‘excluded sequences’ is the number of sequences exceeding the maximum length (default of 300bp), ‘unique sequences’ is the number of unique oligonucleotides generated.

| Library | Generated name | Saturation space | Targeton length | Saturation length | Total sequences | Excluded sequences | Unique sequences |
| --- | --- | --- | --- | --- | --- | --- | --- |
| brca1_nuc_ex2 | chr17_43115634_43115878_minus_sgRNA_ex2 | nucleotide | 245 | 104 | 1000 | 1 | 583 |
| brca1_nuc_ex3 | chr17_43106355_43106599_minus_sgRNA_ex3 | nucleotide | 245 | 128 | 1209 | 1 | 740 |
| brca1_nuc_ex4 | chr17_43104794_43105038_minus_sgRNA_ex4 | nucleotide | 245 | 139 | 1292 | 0 | 825 |
| brca1_nuc_ex5 | chr17_43104080_43104330_minus_sgRNA_ex5 | nucleotide | 251 | 201 | 1439 | 1 | 1185 |
| brca1_pep_ex2 | chr17_43115634_43115878_minus_sgRNA_ex2 | amino acid | 245 | 54 | 340 | 0 | 340 |
| brca1_pep_ex3 | chr17_43106355_43106599_minus_sgRNA_ex3 | amino acid | 245 | 78 | 500 | 0 | 500 |
| brca1_pep_ex4 | chr17_43104794_43105038_minus_sgRNA_ex4 | amino acid | 245 | 89 | 580 | 0 | 580 |
| brca1_pep_ex5 | chr17_43104080_43104330_minus_sgRNA_ex5 | amino acid | 251 | 140 | 920 | 0 | 918 |
